# Whole-Genome Characterization of *Inonotus hispidus* from *Ulmus macrocarpa* and Its Comparative Genomics with Strains from *Morus alba* and *Acer truncatum*

**DOI:** 10.3390/jof11050346

**Published:** 2025-04-29

**Authors:** Ruxue Bai, Qingchun Wang, Haiying Bao

**Affiliations:** 1 Key Laboratory for Development and Utilization of Fungi Traditional Chinese Medicine Resources, Jilin Agricultural University, Changchun 130118, China; bairuxue279@126.com (R.B.); wangqingchun2024@163.com (Q.W.); 2 Key Laboratory of Edible Fungal Resources and Utilization (North), Ministry of Agriculture and Rural Affairs, Jilin Agricultural University, Changchun 130118, China

**Keywords:** *Inonotus hispidus*, *Ulmus macrocarpa*, *Acer truncatum*, whole-genome sequencing, antagonism

## Abstract

*Inonotus hispidus* growing on *Morus alba* is traditionally regarded as the authentic source of the medicinal fungus. However, this species is also found on other host trees, such as *Ulmus macrocarpa* and *Acer truncatum*; yet, whether these strains share comparable genomic and functional traits with *Morus*-derived strains remains unknown. Here, we performed whole-genome sequencing of a strain isolated from *U. macrocarpa* (UMI) using Illumina and PacBio platforms and conducted comparative genomic analysis with strains from *M. alba* (MAI) and *A. truncatum* (AMI). Antagonistic interactions were also evaluated via dual-culture confrontation assays. The UMI genome was 36.44 Mb in size, comprising 9097 predicted genes, of which 6991 and 1672 were annotated in the KEGG and COG databases, respectively. SNP analysis revealed 623,498 and 335,343 variants in AMI and MAI, with AMI showing greater genomic variation. Core–pan genome analysis identified 2651 core genes and 1046, 1424, and 1217 strain-specific genes in UMI, AMI, and MAI, respectively. Phenotypic assays demonstrated distinct mycelial growth dynamics and antagonistic behaviors, which likely reflect host-related environmental adaptation. Overall, *I. hispidus* strains from non-*Morus* hosts exhibit unique genomic and phenotypic features, providing a valuable basis for resource evaluation, artificial domestication, and the medicinal development of wild Sanghuang strains beyond traditional sources.

## 1. Introduction

*Inonotus hispidus* is a significantly large medicinal fungus belonging to the family Hymenochaetaceae, genus *Inonotus* [1,2]. This species is widely distributed across countries such as China, Pakistan, Japan, Germany, Russia, France, the United States, and Algeria [3]. In China, its primary distribution regions include Northeast, East, and Northwest China. *I. hispidus* is an annual fungus that thrives in temperate zones [4] and typically grows on aged *Morus alba* (white mulberry) trees over 40 years old. It is abundantly found in ancient areas along the Yellow River, including Linqing, Xiajin, and Wudi in Shandong Province, as well as Chengde in Hebei Province and Aksu in Xinjiang. In traditional Chinese medicine, it is referred to as “Sanghuang” and has a long history of medicinal use [5]. Beyond mulberry trees, *I. hispidus* also grows on other broad-leaved species such as *Ulmus macrocarpa*, *Acer truncatum*, and *Fraxinus mandshurica* [6]. During the early development of the fruiting body, the primordia appear golden yellow, orange-red, or pale yellow and are typically spherical or irregularly tumor-shaped. As the fruiting body matures, its color gradually darkens from yellow-brown or purple-brown to black [7]. To date, genome sequencing of mononuclear strains of *I. hispidus* growing on *M. alba* and *F. mandshurica* has been conducted, revealing differences in secondary metabolite profiles under different host conditions and the diversity of root-associated endophytic bacteria [8,9,10]. In addition, studies have shown that extracts and monomeric active compounds from the UMI strain exhibit significant inhibitory effects on mammary gland hyperplasia in rat models, primarily by activating the amino acid biosynthesis pathway, with Yakuchinone A identified as the main active compound [11]. However, the whole-genome sequence of the UMI strain remains unavailable, limiting further genetic exploration. To address this gap, we conducted whole-genome sequencing and comparative genomic and biological characterization of the UMI strain.

Since the first eukaryotic genome *Saccharomyces cerevisiae* was successfully sequenced in 1996, fungal genomics has made remarkable progress due to advancements in sequencing technology and declining costs. Compared with other eukaryotes, fungal genomes are relatively simple, facilitating sequence annotation and gene modification. Whole-genome sequencing allows researchers to obtain essential information regarding gene number, structure, coding capacity, metabolic pathways, and signal transduction, which is vital for understanding fungal biology [12]. Comparative genomic analysis across fungal species reveals evolutionary histories, phylogenetic relationships, and gene family expansions or contractions, while also shedding light on ecological adaptability and functional divergence [13]. Moreover, fungal secondary metabolites exhibit diverse bioactivities and pharmacological effects, and, thus, decoding their biosynthetic pathways is crucial for identifying novel drug targets and bioactive molecules such as antibiotics [14]. In recent years, the genomes of several macrofungi have been successfully sequenced, including *Ganoderma lucidum* [15,16], *I. hispidus* [17], *Leucocalocybe mongolica* [18], *Auricularia heimuer* [19], *Russula griseocarnosa* [20], and *Hericium erinaceus* [21]. These studies have deepened our understanding of their gene regulatory networks, metabolic pathways, and medicinal properties, providing a molecular foundation for functional genomics and the systematic mining of secondary metabolites.

Based on this background, we employed the Illumina NovaSeq PE150 and PacBio Sequel platforms to perform whole-genome sequencing of the UMI strain, followed by assembly and gene annotation. Functional predictions were performed using KEGG, CAZy, and other databases to elucidate gene functions and related biological processes. Furthermore, comparative analyses with the MAI and AMI strains—focusing on collinearity, InDels, SNPs, SVs, and core–pan genome structures—were conducted to characterize UMI’s genomic features. Subsequently, antagonism assays among UMI, MAI, and AMI mycelia were conducted to support the proper evaluation of the medicinal potential of wild *I. hispidus* strains from different host tree species and facilitate the comprehensive development and utilization of superior strains. Co-culture assays revealed distinct compatibility and exclusion patterns among the strains, providing a theoretical basis for understanding host specificity and ecological divergence and guiding the selection of elite fungal resources. Given the growing pharmacological interest in terpenoids, polysaccharides, and other bioactive compounds derived from *I. hispidus*, the identification of strains with stable ecological behavior and robust genetic traits may contribute to the targeted development of high-yielding medicinal strains.

## 2. Materials and Methods

### 2.1. Screening and Obtaining Mononuclear Strains of UMI and AMI

**Figure 1:**
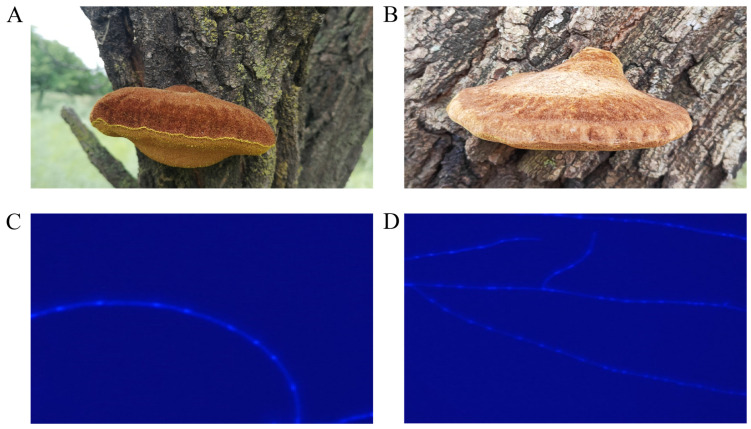
UMI and AMI habitat map and mononuclear strains: (**A**) UMI fruiting body; (**B**) AMI fruiting body; (**C**) UMI mononuclear strains; (**D**) AMI mononuclear strains.

The UMI and AMI strains were collected from the Xianghai National Nature Reserve (45°02′ N, 122°30′ E) in Tongyu County, Baicheng City, Jilin Province, on August 16, 2023, and from the Daqinggou National Nature Reserve (42°58′ N, 122°22′ E) in Horqin Left Back Banner, Tongliao City, Inner Mongolia Autonomous Region, on 17 August 2023, respectively (Figure 1A,B). Spore prints of both strains were obtained using field bagging. The collected spores were serially diluted in sterile water and plated onto PDA medium. Plates were incubated at 30 °C in the dark. Upon the germination of single spores, the resulting colonies were aseptically transferred to fresh PDA plates for subculturing. After 10 days of growth, mycelia were harvested using sterile pipette tips and promptly stored at −80 °C for the preservation of the mononuclear strains.

### 2.2. Genomic DNA Extraction and Whole-Genome Sequencing of UMI and AMI

Genomic DNA was extracted from the mycelia of UMI and AMI using the GP1 extraction method. A 20 kb SMRTbell library and a 350 bp short-fragment library were constructed using the SMRTbell™ Template Prep Kit (version 1.0; Pacific Biosciences, Menlo Park, CA, USA). DNA samples that passed electrophoresis quality control were sheared to the desired fragment size using Covaris g-TUBE (Covaris, Woburn, MA, USA). Following DNA damage repair and end repair, hairpin adaptors were ligated to both ends of the DNA fragments using a DNA ligase. Fragments were then purified and size-selected with AMPure PB magnetic beads. Specific-sized fragments were enriched, and shorter fragments were removed using the BluePippin system (Sage Science, Beverly, MA, USA). The concentration of the constructed library was measured using a Qubit fluorometer (Thermo Fisher Scientific, Waltham, MA, USA) and the fragment size distribution was assessed with an Agilent 2100 Bioanalyzer (Agilent Technologies, Santa Clara, CA, USA). Sequencing was performed using PacBio third-generation, single-molecule, real-time (SMRT) technology (Pacific Biosciences, Menlo Park, CA, USA) combined with Illumina second-generation, high-throughput sequencing (Illumina, San Diego, CA, USA) [22]. The sequencing data were assembled with Hifiasm v0.16.1 to generate the complete genome map. Circos v0.69.6. software was used to visualize the genomic architecture [23].

### 2.3. Genome Prediction and Functional Annotation

Glimmer, Prodigal v2.6.3, and GeneMarkS v4.3 software were used to predict the coding genes of the assembled genome sequence. The ribosomal RNA (RNA) gene was predicted by Barrnap v0.9 software and the transfer RNA (RNA) gene was predicted by tRNAscan-SE v2.0.12 software. In addition, GO (Gene Ontology, http://geneontology.org/, accessed on 20 February 2025) [24], KEGG (Kyoto Encyclopedia of Genes and Genomes, http://www.genome.jp/kegg/, accessed on 20 February 2025) [25], KOG (evolutionary genealogy of genes: Non-supervised Orthologous Groups) [26], and CAZy (Carbohydrate-Active enZymes database, http://www.cazy.org/, accessed on 20 February 2025) [27] were used for functional annotation. The predicted genes were compared against the TCDB database (http://www.tcdb.org/, accessed on 20 February 2025) [28] using blastp (e-value ≤ 1 × 10^^−5^). Other general functional databases were also used for annotation. The secreted protein and secondary metabolite gene clusters were predicted using SignalP (http://www.cbs.dtu.dk/services/SignalP/, accessed on 20 February 2025) [29] and antiSMASH v6.1(https://antismash.secondarymetabolites.org/, accessed on 20 February 2025) [30] databases.

### 2.4. Comparative Genomic Analysis

The genome of MAI (BioProject: PRJNA973857; BioSample: SAMN35152529; SRA: SUB13375186) was selected for comparative analysis between UMI and AMI. The AMI genome was sequenced exclusively using the Illumina platform. Pairwise genome alignments among the three strains were performed using MUMmer v4 [31] to identify syntenic regions. Alignment results were further refined using LASTZ v1.04.03 [32] to detect structural rearrangements, including translocations (Trans), inversions (Inv), and composite events (Trans + Inv). Variants including SNPs, InDels, and SVs were identified from the alignment outputs and filtered to ensure accuracy. For core and pan-genome analysis, gene sets were compared using BLAST+ v2.12.0. The Blast Coverage Ratio (BCR) was calculated using the following formulas [33]: BCR (Ref) = (Match/Length (R)) × 100%; BCR (Que) = (Match/Length (Q)) × 100%. Genes with reciprocal BCR values exceeding the defined thresholds were classified as core genes; others were categorized as accessory or strain-specific genes, collectively forming the pan-genome.

### 2.5. Biological Characterization of UMI, AMI, and MAI

Determination of mycelial growth rates: The PDA basal medium was prepared using 200 g of peeled potato, 20 g of anhydrous glucose, 18 g of agar, and 1000 mL of distilled water. The medium was sterilized at 121 °C and 1 × 10^5^ Pa for 30 min. UMI, AMI, and MAI strains preserved at 4 °C on slanted solid media were reactivated by culturing on PDA plates at 30 °C in a constant-temperature incubator. After visible hyphal growth, mycelial plugs (0.8 cm in diameter) with similar growth rates were excised and inoculated onto fresh PDA plates under a sterile workbench. Each treatment was performed with five biological replicates. Starting from the second day post-inoculation, colony diameters were measured daily using the cross–cross method at fixed time points. Colony morphology and mycelial growth were recorded until the fastest-growing colony was nearly covering the plate.

Antagonistic interaction assays: For confrontation experiments, mycelial plugs of UMI, AMI, and MAI were again inoculated onto PDA medium using a sterile punch under an ultra-clean workbench. Plates were incubated at 30 °C in the dark to observe antagonistic relationships among the strains.

## 3. Results and Discussion

### 3.1. Identification of Mononuclear Strains from UMI and AMI

UMI and AMI hyphae stained with DAPI were observed using an Axio Imager A2 fluorescence microscope(Carl Zeiss, Oberkochen, Germany), and image processing was performed using Dimension software v4.0 (Carl Zeiss, Oberkochen, Germany). The results showed that the mycelia of both strains were mononuclear. The UMI strain exhibited strong mycelial fluorescence, with a diameter of approximately 3–4 μm, relatively concentrated nuclear distribution, and uniform nuclear morphology (Figure 1C). In contrast, the AMI strain displayed weaker fluorescence, with a mycelial diameter of approximately 2–3 μm, more dispersed nuclear distribution, and larger nuclear spacing in certain regions (Figure 1D). Quantitative analysis using image analysis software indicated that the fluorescence intensity of the UMI strain was significantly higher than that of the AMI strain (UMI: 1300 ± 60 A.U.; AMI: 1000 ± 45 A.U.; *p* < 0.05). The more concentrated nuclear distribution observed in the UMI strain may have been related to differences in the mycelial structural features.

### 3.2. Genome Assembly and General Features of UMI

**Figure 2:**
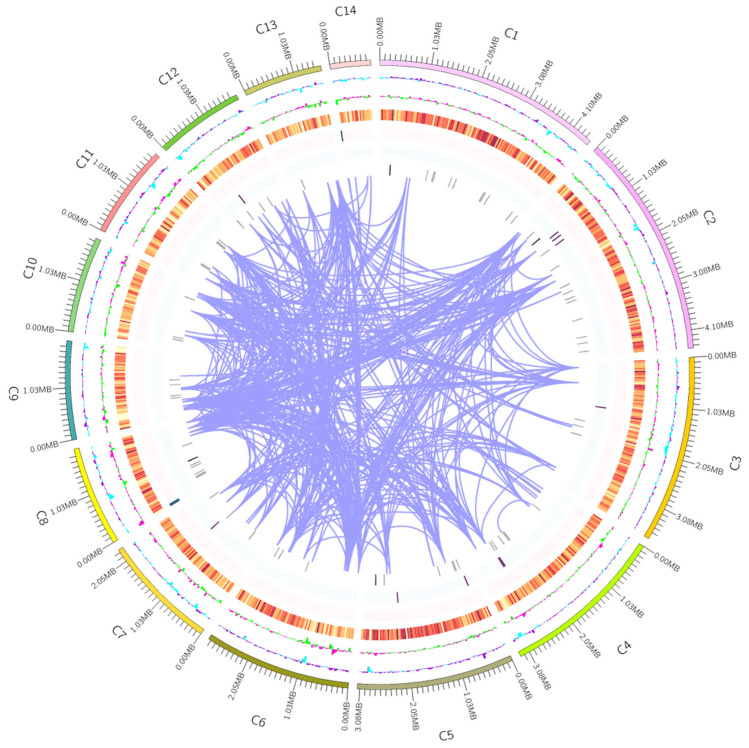
Genome-wide map of UMI. Note: The outermost circle is the position coordinate of the genome sequence. From the outside to the inside is the GC content of the genome. The GC content is counted by the window (genome/1000) bp and the step size (genome/1000) bp. The blue part indicates that the GC content in the region was lower than the average GC content of the whole genome. The purple part shows the opposite, and the higher the peak, the greater the difference from the average GC content. The genome GC skew value: window (genome/1000) bp, step size (genome /1000) bp, the specific algorithm was (G − C) / (G + C). The inward green part indicates that the content of G in this region was lower than that of C, and the outward pink part shows the opposite gene density (the gene density of the coding gene and rRNA, snRNA, and tRNA was calculated by window genome/1000 bp and step genome/1000 bp, respectively; the darker the color, the greater the gene density in the window) and chromosome duplication.

The genome of UMI was de novo assembled using a combination of PacBio long-read and Illumina short-read sequencing data. After adapter trimming and the removal of low-quality reads, high-quality PacBio reads were error-corrected using Illumina data, resulting in a high-fidelity genome assembly (Figure 2). The final assembly yielded a genome size of 36.44 Mb, consisting of 15 contigs with a GC content of 48.39%. The longest contig was 48,990 bp and the shortest was 144 bp. The assembly achieved an N50 of 3.12 Mb and an average sequencing depth of approximately 200×. Completeness assessment using BUSCO v5.4.3 indicated a score of 99.5%, reflecting excellent genome integrity and assembly quality (Table 1). Comparative analysis with published genomes of *Inonotus obliquus* [34,35], *Sanghuangporus vaninii* [36], and *S. sanghuang* [37,38] further supported the high quality of the UMI genome. Its genome size, GC content, and completeness were comparable to or exceeded those of related medicinal fungi, confirming that the UMI genome provides a robust reference for downstream applications such as gene prediction, functional annotation, and comparative genomics.

### 3.3. Genome-Wide Functional Annotation of UMI

#### 3.3.1. General Functional Annotation Overview

**Figure 3:**
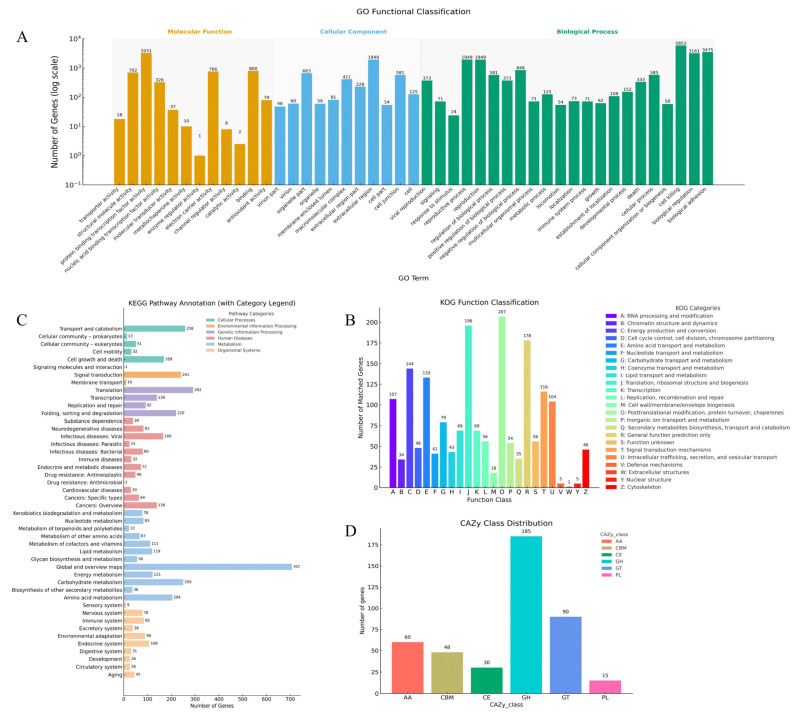
Genome function annotation of UMI: (**A**) GO; (**B**) KOG; (**C**) KEGG; (**D**) CAZy.

Functional annotation of the UMI genome based on four major databases—GO, KOG, KEGG, and CAZy—revealed a strong enrichment of genes associated with metabolism, catalytic activity, and cellular structure. A total of 5852 genes were annotated in the Gene Ontology (GO) database, with the most represented categories being metabolic processes and catalytic activity (Figure 3A). In the KOG database, 1672 genes were assigned to 24 functional groups, with post-translational modification and metabolic processes as the most enriched categories (Figure 3B). KEGG pathway analysis mapped 4687 genes to 369 pathways, of which 1854 genes (39.6%) were involved in essential metabolic pathways. Notably, pathways related to carbohydrate, amino acid, energy, and lipid metabolism were prominently enriched, suggesting that UMI possesses substantial metabolic plasticity (Figure 3C). CAZy annotation identified 423 carbohydrate-active enzymes (CAZymes), including 185 glycoside hydrolases (GHs) and 60 auxiliary activity enzymes (AAs), many of which are known to be involved in lignin and cellulose degradation (Figure 3D). Collectively, these results indicate that *I*. *hispidus* UMI has extensive genomic potential for primary metabolism, carbon utilization, and environmental adaptation.

#### 3.3.2. KEGG Pathway Annotation and Core Metabolic Processes

**Figure 4:**
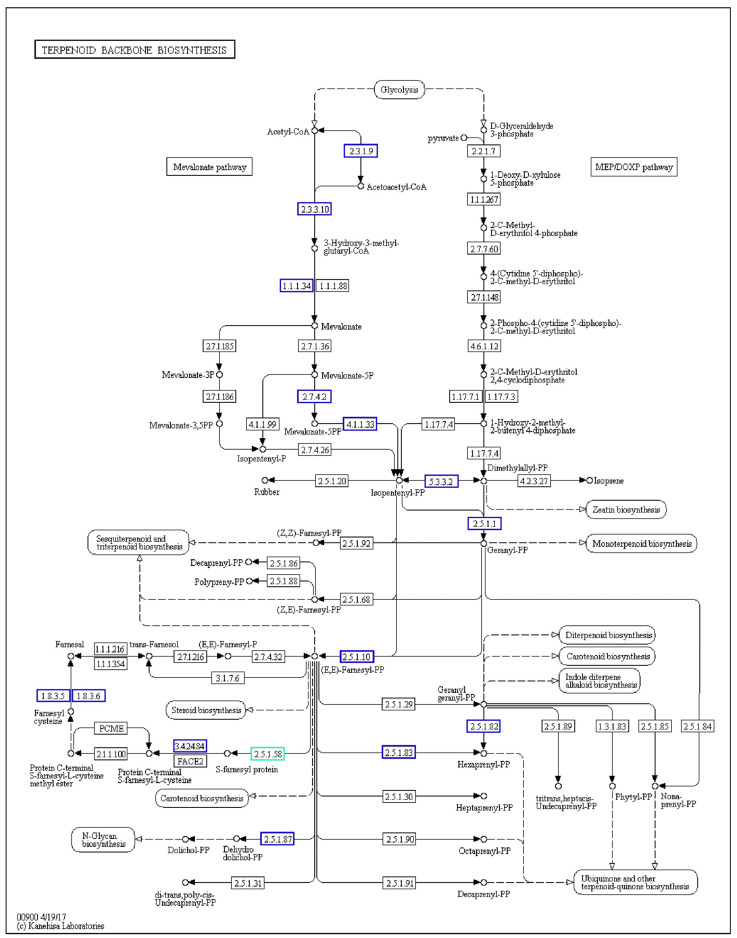
Key enzymes involved in “terpenoid backbone biosynthesis (map 00900)” in UMI. Note: The blue box indicates that the homologous gene of the enzyme exists, the green box indicates that the homologous gene of the enzyme repeats, and the white box indicates that it does not exist. Analysis was performed using KEGG Mapper (https://www.genome.jp/kegg/mapper/, accessed on 22 February 2025).

KEGG annotation of the UMI genome revealed 4687 protein-coding genes mapped to 369 metabolic pathways, categorized into six major functional groups. Among them, 1854 genes (39.6%) were involved in primary metabolism, highlighting UMI’s strong capacity for essential physiological functions. Notably, genes associated with carbohydrate (250 genes), amino acid (204 genes), energy (121 genes), cofactor and vitamin (111 genes), and lipid (119 genes) metabolism were particularly enriched, reflecting their adaptation to woody substrates and broad metabolic potential (Figure 3C). These features are consistent with patterns observed in other wood-decaying fungi such as *Lentinula edodes* and *Ganoderma lucidum* [39,40]. Further analysis of the terpenoid backbone biosynthesis pathway (KEGG map00900) revealed 13 key enzyme-coding genes, including hexaprenyl–diphosphate synthase, farnesyl diphosphate synthase, and protein farnesyltransferase/geranylgeranyltransferase type-1 subunit alpha, with certain genes occurring in multiple copies (Table 2, Figure 4). Additionally, a gene encoding lanosterol synthase (LSS, ERG7; EC 5.4.99.7) was identified, which catalyzes the cyclization of oxidosqualene to lanosterol, a central precursor of steroids and triterpenoids. These findings suggest that UMI possesses a complete enzymatic toolkit for triterpenoid biosynthesis, with potential for industrial application through the microbial production of lanosterol or ergosterol.

**Figure 5:**
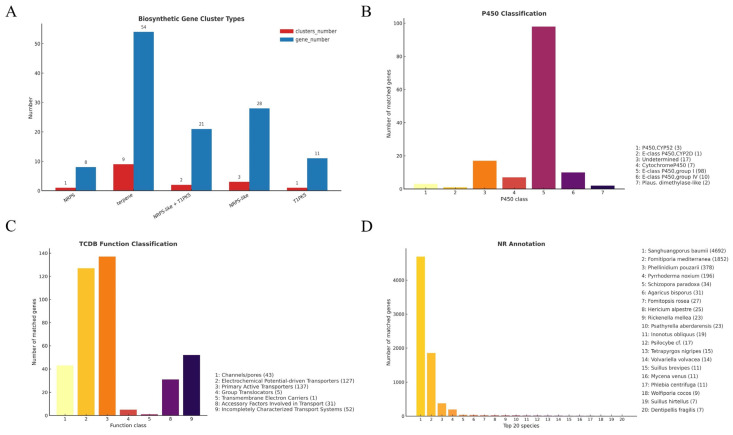
Specific genome function annotation: (**A**) secondary metabolism gene clusters; (**B**) CYP450; (**C**) TCDB; (**D**) NR.

In addition, a total of 16 secondary metabolite biosynthetic gene clusters (SMGCs) were predicted in the UMI genome using antiSMASH v6.1, including 9 terpene clusters (comprising 54 genes), 3 NRPS-like clusters, 2 hybrid NRPS–T1PKS clusters, 1 T1PKS cluster, and 1 NRPS cluster (Figure 5A). The abundance of terpene gene clusters was consistent with the KEGG-based identification of key enzymes in the terpenoid biosynthetic pathway, particularly lanosterol synthase (ERG7), suggesting an active triterpene biosynthesis system [41]. These clusters are typically responsible for the biosynthesis of structurally diverse natural products with antimicrobial, antitumor, and immunomodulatory activities [42,43]. The co-occurrence of these pathway enzymes and biosynthetic gene clusters reinforces the notion that UMI has strong biosynthetic potential. Similar gene clusters have been reported in *Inonotus obliquus* and other basidiomycetes, where secondary metabolism often exhibits strain-specific expression patterns and is tightly regulated [44,45]. Although experimental validation of these genes is still needed, the genomic architecture of UMI provides a robust foundation for the further exploration of secondary metabolite biosynthesis and regulatory mechanisms.

#### 3.3.3. CAZyme Families and Lignocellulose Degradation Potential

Carbohydrate-active enzymes (CAZymes) are essential for the degradation of plant-derived polymers and play a central role in fungal ecological adaptation. As a white-rot fungus, *I. hispidus* can decompose lignocellulose efficiently, contributing to biomass recycling and carbon cycling in forest ecosystems. Lignocellulose degradation typically requires the synergistic action of CAZymes and oxidoreductases, with the former being key tools for fungi to acquire carbon sources in woody environments. In the UMI genome, 423 CAZyme genes were identified, classified into six major families: glycoside hydrolases (GHs, 185), glycosyltransferases (GTs, 92), auxiliary activities (AAs, 60), carbohydrate esterases (CEs, 29), carbohydrate-binding modules (CBMs, 48), and polysaccharide lyases (PLs, 9). These enzymes span 115 CAZy families, reflecting enzymatic diversity. Cellulases and hemicellulases were found primarily in GH1–GH3, GH5, GH6, GH9, and CE families. Pectinases were distributed across CE, GH, and PL families. Ligninolytic enzymes, including laccases and peroxidases, were enriched in AA1 and AA2. The classification and abundance of CAZyme families are presented in Figure 3D.

These enzymatic features indicate UMI’s strong adaptation to complex plant cell walls. The type and number of CAZymes are tightly correlated with fungal lifestyle and substrate preference [46]. Similar patterns are observed in fungi such as *Pleurotus ostreatus* and *Lentinula edodes*, which also possess expanded CAZy repertoires [47,48]. Lignocellulose degradation by UMI is thus mediated by a broad CAZyme arsenal, functioning in synergy with oxidoreductases. These findings establish a foundation for the further exploration of lignin degradation mechanisms and open avenues for developing UMI strains for biomass utilization and biotechnological applications [49].

#### 3.3.4. Cytochrome P450 Gene Family Analysis

Cytochrome P450 monooxygenases (CYP450s) are essential oxidoreductases involved in fungal growth, metabolism, and secondary biosynthesis. A total of 136 CYP450 genes were identified in the UMI genome, accounting for 0.42% of predicted genes. The majority were classified into E-class P450 group I (96 genes) and group IV (10 genes). Notably, the CYP51 family was present, known to catalyze the 14α-demethylation of lanosterol during ergosterol biosynthesis, a key structural component of fungal membranes. Additionally, a gene encoding lanosterol synthase (ERG7; EC 5.4.99.7) was identified, which cyclizes oxidosqualene into lanosterol, a precursor of steroids and triterpenoids. These findings suggest that UMI possesses a complete steroid biosynthesis pathway, supporting its metabolic diversity and industrial potential. Functional enrichment analysis further revealed that 19 genes were associated with the KEGG sub-pathway “Metabolism of xenobiotics by cytochrome P450” and 17 genes with “Drug metabolism-cytochrome P450,” indicating roles in detoxification and environmental stress adaptation. The classification and KEGG mapping of CYP450-related genes are presented in Figure 5A.

Comparative studies have shown that macrofungi tend to possess large CYP450 repertoires, with *Ganoderma lucidum* encoding 219 CYP450 genes across 42 families [50], and other medicinal fungi such as *Antrodia cinnamomea* (119 genes), *Hypsizygus marmoreus* (132 genes), and *Hericium erinaceus* (137 genes) displaying similar CYP450 diversity [42,43,47]. The number and family distribution of CYP450 genes in UMI are consistent with these fungi, reflecting its potential to synthesize structurally diverse natural compounds such as terpenoids, steroids, and alkaloids. The diversity of CYP450s underscores their central role in the biosynthetic plasticity of *I. hispidus* and its medicinal relevance.

#### 3.3.5. Membrane Transporters Annotated via TCDB

Membrane transport proteins are essential for substrate uptake, metabolite secretion, and stress response in fungi. Based on TCDB annotation (Figure 5B), a total of 396 transporter genes were identified in the UMI genome, indicating substantial transporter diversity. The majority were classified as primary active transporters (e.g., ABC transporters) and electrochemical potential-driven transporters (e.g., MFS transporters), both of which are associated with energy-dependent substrate translocation. These systems are known to mediate multidrug resistance, the secretion of secondary metabolites, and nutrient acquisition. Other transporter classes included channel proteins, accessory factors, and a notable portion of poorly characterized systems, suggesting potential for novel transporter mechanisms. Similar transporter profiles have been reported in other medicinal fungi such as *Ganoderma lucidum* and *Lentinula edodes*, highlighting the central role of membrane transporters in fungal environmental adaptation and biosynthetic regulation [48,50].

#### 3.3.6. Overall Annotation Coverage and Species-Level Homology

Although extensive annotation of the UMI genome was performed using multiple public databases, a substantial proportion of genes remained functionally unclassified. KEGG annotation identified 4687 genes (21.45% of predicted coding sequences), while Swiss-Prot annotated 2199 genes (6.74%). Despite being relatively low, these rates are consistent with other macrofungi sequenced using third-generation platforms, such as *Lentinula edodes* (17.4%) and *L. mongolica* (29.1%) [18]. This limited coverage reflects known challenges in fungal genome annotation, especially in Basidiomycetes, which often harbor lineage-specific genes and lack representation in curated protein databases [51]. These findings underscore the need to integrate transcriptomic and proteomic data in future analyses to improve annotation depth and accuracy. Nevertheless, we focused subsequent analyses on well-annotated gene regions to explore core functions related to metabolism, development, and host interaction.

In addition, NR (non-redundant protein) database comparisons revealed 7702 UMI genes with homologs in characterized species. The top three matched taxa were *Sanghuangporus baumii* (4692 genes), *Fomitiporia mediterranea* (1852 genes), and *Phellinidium pouzarii* (378 genes), all of which are wood-decaying or medicinal basidiomycetes. These results indicate strong phylogenetic affinity between UMI and ligninolytic fungi with similar ecological roles. The high level of sequence homology suggests potential functional convergence in substrate utilization and secondary metabolite biosynthesis. These patterns, summarized in Figure 5D, highlight UMI’s placement within the Hymenochaetaceae clade and support its value as a model for studying host-adaptive metabolism and fungal evolution [52].

These homology patterns are further supported by the functional characteristics observed in UMI’s annotated gene families. The presence of diverse CAZymes, particularly those involved in lignocellulose degradation (e.g., GHs, AAs), aligns with the enzymatic repertoire of *S. baumii* and *F. mediterranea*, reflecting convergent ecological strategies [53]. Similarly, the size and diversity of the CYP450 gene family in UMI mirror those of other ligninolytic or medicinal fungi, suggesting conserved oxidative mechanisms for both primary and secondary metabolism [52]. The membrane transporter systems, especially ABC and MFS families, and the extensive secondary metabolite biosynthetic gene clusters (SMGCs) further corroborate UMI’s metabolic versatility and its potential for natural product biosynthesis. Taken together, these findings underscore the evolutionary and functional parallels between UMI and its phylogenetically related species, reinforcing its value as a model for studying host-adapted metabolism and fungal biotechnological potential [41].

### 3.4. Comparative Genomic Analysis of UMI, AMI, and MAI Strains

#### 3.4.1. Comparison Results of Whole-Genome Sequencing

Among the three strains of *I. hispidus*, UMI exhibited the largest genome and highest number of predicted protein-coding genes, suggesting a possible genome expansion. In contrast, AMI had the smallest genome and gene number, accompanied by a higher GC content. These differences may reflect strain-specific adaptation to distinct host tree species. All three genomes contained typical non-coding RNAs, supporting overall completeness (Table 3; Supplementary Table S1). The relatively higher GC content observed in AMI may contribute to increased genomic stability or altered transcriptional dynamics, which could be linked to its distinctive physiological features. These findings provide a genomic basis for the observed phenotypic and ecological divergence among the strains and lay a foundation for further functional analysis.

#### 3.4.2. Genome Collinearity and Rearrangement

**Figure 6:**
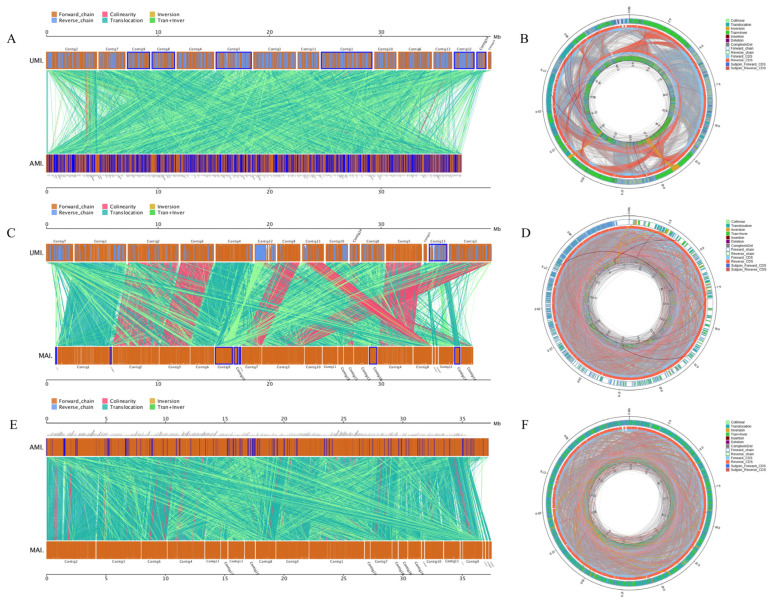
Collinearity analysis and SV results: (**A**) collinearity analysis of MAI vs. UMI; (**B**) SV analysis of MAI vs. UMI; (**C**) collinearity analysis of AMI vs. MAI; (**D**) SV analysis of AMI vs. UMI; (**E**) collinearity analysis of AMI vs. MAI; (**F**) SV analysis of AMI vs. MAI.

Whole-genome collinearity analysis revealed strong structural conservation among the three *I*. *hispidus* strains, despite the presence of detectable variations such as chromosomal inversions and translocations. UMI and MAI exhibited the highest degree of collinearity (86.66%, 14,368 syntenic blocks), whereas AMI showed more extensive genome rearrangement and reduced synteny with UMI (83.87%, 10,304 blocks), indicating enhanced genomic plasticity (Figure 6A,C,E). These findings suggest that AMI may have undergone accelerated genome restructuring in response to host-specific environmental pressures. Similar host-associated genomic reorganization has been reported in other wood-decaying basidiomycetes, including *Fomitiporia mediterranea* and *Sanghuangporus sanghuang*, where long-term host adaptation correlates with extensive synteny disruption and chromosomal reshuffling [37]. Despite such divergence, the presence of large, conserved, syntenic regions across all three strains points to a stable core genome architecture and shared functional modules, reflecting both evolutionary ancestry and functional constraint. However, it remains unclear whether the observed structural rearrangements disrupt or reposition critical genomic features such as secondary metabolite gene clusters, regulatory regions, or transcription factor binding sites. In related basidiomycetes, such as white-rot fungi, chromosomal inversions and translocations have been associated with the altered expression of genes involved in lignin degradation and antifungal metabolite biosynthesis [51]. Therefore, future comparative transcriptomic and epigenomic studies are warranted to determine whether these genome-scale structural changes influence expression patterns, regulatory network architecture, and, ultimately, the ecological and metabolic capacity of *I. hispidus* strains.

#### 3.4.3. Indel, SNP, and SV Analysis

Comparative genomic analysis revealed a markedly higher accumulation of small and large sequence variants in AMI compared to MAI. AMI harbored 8356 insertion–deletion events (InDels), including 1392 in coding regions, whereas MAI exhibited only 177 coding-region InDels. Among these, AMI contained a notably higher number of frameshift and premature stop mutations, suggesting increased disruption of gene function and possible pseudogenization events. Single-nucleotide polymorphism (SNP) analysis reinforced this trend: AMI exhibited nearly twice the number of SNPs as MAI (623,498 vs. 335,343), with a higher proportion occurring within coding sequences (179,844 vs. 90,835). The predominance of nonsynonymous and loss-of-function variants in AMI suggests a higher mutational load and potentially accelerated sequence evolution.

Structural variant (SV) analysis revealed a similar pattern (Figure 6B,D,F). AMI exhibited 9096 SVs—including 3446 complex events involving inversions and translocations—compared to 3491 SVs in MAI. These findings indicate widespread chromosomal instability in AMI and suggest ongoing genomic remodeling. This level of structural plasticity is consistent with prior observations in other host-adapted fungi, such as *Agaricus bisporus* and *Lentinula edodes*, where host-specific pressures and substrate variability have been linked to elevated mutational rates and adaptive genome reshaping [54]. Importantly, the genomic divergence between AMI and the other strains aligns with their observed phenotypic differences, particularly in antagonistic behavior and growth dynamics. These patterns support a model in which host-associated environmental pressures not only drive genomic divergence but also contribute to functional specialization in *I. hispidus*. Further investigation is needed to determine which gene families—such as transcription factors, signaling proteins, or secondary metabolism enzymes—are disproportionately affected by disruptive mutations. The integration of transcriptomic profiling under varying host conditions may clarify whether these mutations translate into differential gene expression patterns that underpin strain-specific ecological adaptations.

#### 3.4.4. Core-Pan Genome Analysis

**Figure 7:**
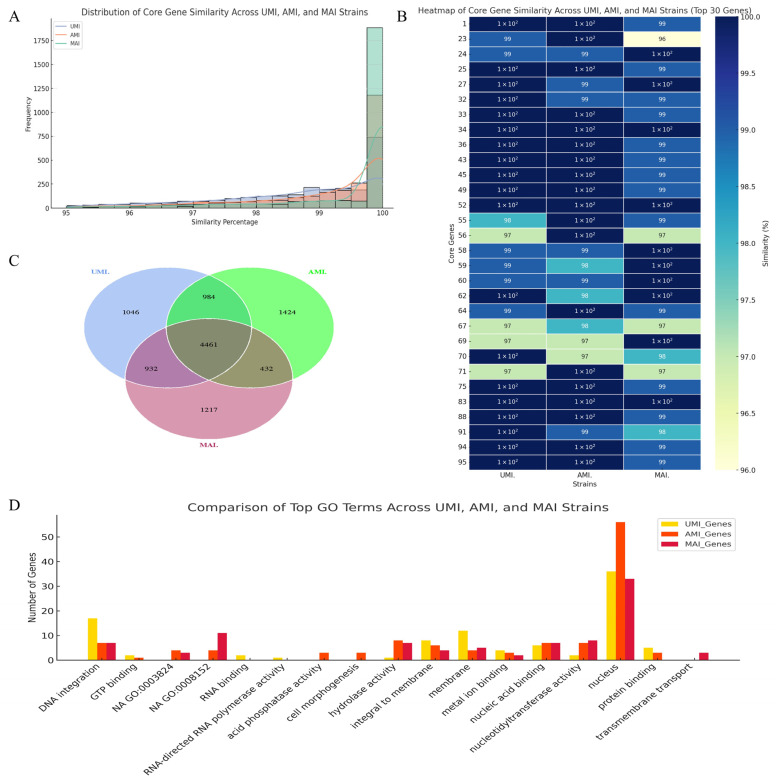
Core–pan genome and functional enrichment analysis of *I. hispidus* strains: (**A**) core gene statistics; (**B**) similarity heatmap of the top 30 core genes; (**C**) Venn diagram of strain-specific genes; (**D**) functional enrichment of unique genes.

Core–pan genome analysis of the three *I*. *hispidus* strains (UMI, AMI, and MAI) identified 25,819 orthologous gene clusters, comprising 10,496 pan genes (40.65%), 4461 core genes (17.28%), and 6035 dispensable genes (23.37%). In addition, each strain contained a unique set of strain-specific genes: 1147 in UMI, 1494 in AMI, and 1271 in MAI (Figure 7A–C), reflecting substantial intraspecific genomic variation. Functional enrichment of strain-specific genes revealed pronounced divergence in biological roles. Protein binding was the most overrepresented function across all strains, with AMI having the highest number of associated genes (57 genes). Nucleic acid binding and hydrolase activity were common among strains, each contributing eight specific genes in both AMI and MAI. Genes involved in DNA integration were exclusively present in UMI (17 genes) and MAI (7 genes) but completely absent in AMI, indicating a possible reduction in transposon activity or recombination-related processes. Membrane-associated functions (“integral to membrane” and “membrane”) were enriched in all strains, with UMI showing the highest number of genes in this category, suggesting its potential specialization in host–fungus interface interactions. In contrast, AMI-specific genes were enriched in RNA polymerase activity and RNA-directed functions, whereas MAI-specific genes were associated with nuclear processes and transmembrane transport (Figure 7D).

These findings mirror observations in other symbiotic basidiomycetes, where strain-level gene divergence is closely linked to host specificity and ecological adaptation [53,55]. The functional partitioning observed among UMI, AMI, and MAI likely reflects divergent evolutionary trajectories shaped by host-associated environmental pressures. Notably, the expansion of regulatory genes in AMI may enhance transcriptional flexibility under variable host conditions, whereas UMI’s enrichment in membrane-related genes suggests an increased capacity for environmental sensing and signaling. Future studies integrating transcriptomic profiling and functional genomics (e.g., gene knockout, complementation, or overexpression) will be critical for elucidating the roles of these strain-specific gene sets in driving ecological specialization and phenotypic divergence in *I. hispidus*.

### 3.5. Comparative Analysis of Phenotypic Characteristics of UMI, AMI, and MAI Strains

**Figure 8:**
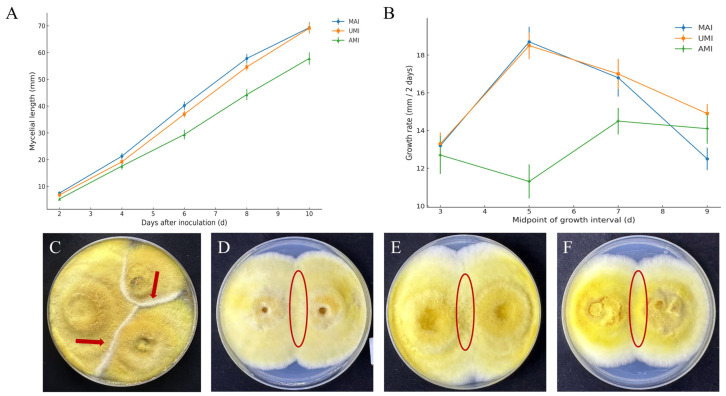
Mycelial growth and antagonism test results. (**A**) Mycelial growth of MAI, UMI, and AMI strains cultured on basal medium. Data are presented as mean ± SD (n = 5). (**B**) Mycelial growth rates of MAI, UMI, and AMI strains, calculated at 2-day intervals, with the X-axis representing the midpoint of each interval. Values represent mean ± SEM (n = 5). (**C**) UMI–MAI–AMI: arrows indicate the antagonistic lines formed between colonies. (**D**) UMI–UMI; (**E**) MAI–MAI; and (**F**) AMI–AMI: circles indicate areas of mycelial fusion between colonies.

A comparative analysis was performed to assess the mycelial growth of the MAI, UMI, and AMI strains. All strains exhibited normal growth on basal medium, with no apparent differences in colony morphology. However, quantitative measurements revealed distinct growth dynamics among the strains (Figure 8A). To further characterize these differences, a segmented analysis of mycelial growth rates was conducted. The results showed no statistically significant difference between MAI and UMI (*p* > 0.05), whereas AMI exhibited significantly lower growth rates than both (*p* < 0.05) (Figure 8B).

To investigate potential antagonistic interactions, dual-culture plate confrontation assays were conducted among the MAI, UMI, and AMI strains. Distinct inhibition lines were observed at the hyphal junctions, accompanied by brown pigment deposition on the reverse side of the plates, suggesting a degree of incompatibility or competitive exclusion among the strains (Figure 8C). In contrast, control groups composed of identical strains (UMI–UMI, AMI–AMI, MAI–MAI) exhibited seamless hyphal fusion and no antagonistic response, indicating that the observed interactions were not artifacts of environmental or cultural conditions (Figure 8D–F). These findings suggest that *I*. *hispidus* strains derived from different host trees may exhibit ecological niche differentiation or underlying genetic divergence, warranting further functional classification and resource evaluation. The phenotypic differences observed are consistent with the genomic divergence identified in prior comparative analyses. The enhanced late-stage growth of UMI may reflect strain-specific regulatory adaptations, such as altered transcriptional timing or increased substrate utilization efficiency, potentially governed by unique gene clusters involved in carbon metabolism or membrane transport [49]. Conversely, AMI’s persistently lower growth rate may be attributed to variations in genes related to energy metabolism or CAZyme (carbohydrate-active enzyme) activity, consistent with its higher SNP density and the presence of strain-specific genes [56]. Moreover, the clear antagonistic interactions observed in non-identical pairings—evidenced by inhibition lines and pigment accumulation—further support the presence of competitive exclusion mechanisms mediated by secondary metabolites. This interpretation aligns with previous studies suggesting that such interactions are frequently associated with biosynthetic gene clusters encoding terpenoids, polyketides, and other potentially antimicrobial compounds [57,58]. Collectively, these findings confirm functional divergence at the genomic level and suggest that long-term host association may drive ecological specialization in *I. hispidus*. The observed inter-strain differences highlight the importance of considering host ecology and genotype–environment interactions in the breeding and development of edible and medicinal fungi [59].

### 3.6. Genomic Features and Application Potential of I. hispidus Strains

This study presents an in-depth genomic characterization of the UMI strain of *I*. *hispidus*, highlighting its extensive capacity for carbon metabolism, oxidative processes, and secondary metabolite biosynthesis. The identification of terpene synthases, polyketide synthases, cytochrome P450s, and a broad array of carbohydrate-active enzymes and membrane transporters underscores the metabolic versatility of UMI, providing a strong foundation for future applications in bioactive compound production, biodegradation, and host-adaptive signaling. Although the AMI genome was not fully analyzed due to limitations in sequencing depth and assembly contiguity—its complete annotation is provided in the Supplementary Materials—comparative analyses nonetheless revealed key genomic and phenotypic traits of this strain. AMI exhibited the highest density of SNPs and structural variants among the three strains, suggesting accelerated sequence evolution and potential niche-specific adaptation. Moreover, AMI-specific genes were enriched in transcriptional regulation and energy metabolism pathways, potentially correlating with its distinct growth profile and enhanced stress tolerance. These results suggest that AMI represents a valuable yet underexplored genetic resource, meriting further investigation using higher-resolution genomic and transcriptomic approaches.

Comparative analyses also demonstrated that while MAI, the traditionally favored strain derived from *Morus alba*, maintains advantages in early-stage growth and phenotypic stability, UMI and AMI exhibit broader genomic plasticity and potential for metabolic diversification. The observed inter-strain differences in ecological adaptation, antagonistic behavior, and biosynthetic capacity reflect divergent evolutionary pressures shaped by host environments, supporting the hypothesis of host-driven genome specialization in *I. hispidus*. Collectively, this study not only elucidates the genomic architecture of a representative *I. hispidus* strain but also identifies promising alternative strains—such as UMI and AMI—that may serve as complementary or replacement resources for future industrial and medicinal applications. These findings lay a theoretical and technical foundation for the selective breeding, functional gene discovery, and precision engineering of *I. hispidus* as a model organism in fungal biotechnology.

## 4. Conclusions

The genome sequence of the *I. hispidus* UMI strain, isolated from *Ulmus macrocarpa*, provides a valuable foundation for investigating the genetic basis and biological functions of this edible and medicinal fungus. The identified coding genes and metabolic pathways enhance the understanding of UMI’s functional characteristics and establish a basis for exploring the biosynthetic mechanisms of its bioactive metabolites. Comparative genomic analyses revealed that although strains from different hosts belong to the same species, their genomes exhibit substantial divergence, likely associated with host-specific interactions and antagonistic behaviors. In future studies, special attention should be given to terpene biosynthetic gene clusters, which are highly enriched in UMI, as they may offer critical insights into the synthesis and regulation of secondary metabolites. Although some predicted genes associated with phenotypic traits have not yet been experimentally validated, the high-quality genome data of UMI presented in this study provide a theoretical and technical foundation for the development and utilization of wild *I. hispidus* medicinal resources, as well as for the screening and domestication of superior strains.

## Figures and Tables

**Table 1:** Genome-wide feature information of UMI.

Statistics	UMI
Genome size (bp)	36,444,366
Gene number	9097
Gene total length (bp)	13,783,881
Gene average length (bp)	1515
Gene length/genome (%)	37.82
Gene internal length	22,660,485
Gene internal GC content	46.4
tRNA	111
snRNA	10
LTR	2519
DNA	555
LINE	392
SINE	11
RC	35
TR	3363
Minisatellite DNA	2279
Microsatellite DNA	615

**Table 2:** Key enzymes involved in “terpenoid backbone biosynthesis (map 00900)” of UMI.

Gene ID	KO Term	Gene Name and Definition	EC Number
A2154	hexPS, COQ1	hexaprenyl-diphosphate synthase	2.5.1.82 2.5.1.83
A2770	idi, IDI	isopentenyl-diphosphate Delta-isomerase	5.3.3.2
A2861	PCYOX1, FCLY	prenylcysteine oxidase/farnesylcysteine lyase	1.8.3.5 1.8.3.6
A3529	DHDDS, RER2, SRT1	ditrans, polycis-polyprenyl diphosphate synthase	2.5.1.87
A3653	HMGCR	hydroxymethylglutaryl-CoA reductase (NADPH)	1.1.1.34
A4362	FDPS	farnesyl diphosphate synthase	2.5.1.1 2.5.1.10
A4588	E2.3.3.10	hydroxymethylglutaryl-CoA synthase	2.3.3.10
A5296	MVD, mvaD	diphosphomevalonate decarboxylase	4.1.1.33
A5897	E2.7.4.2, mvaK2	phosphomevalonate kinase	2.7.4.2
A6225	E2.3.1.9, atoB	acetyl-CoA C-acetyltransferase	2.3.1.9
A8924	FNTA	protein farnesyltransferase/geranylgeranyltransferase type-1 subunit alpha	2.5.1.58 2.5.1.59
A0619	STE24	STE24 endopeptidase	3.4.24.84
A0708	FNTB	protein farnesyltransferase subunit beta	2.5.1.58

**Table 3:** Comparison results of basic genetic characteristics of UMI, AMI, and MAI.

Statistics	Sample		
	UMI	AMI	MAI
Genome size (bp)	36,444,366	33,809,908	34,136,716
Gene number	9097	8366	8356
Gene total length (bp)	13,783,881	11,802,855	12,839,852
Gene average length (bp)	1515	1411	1537
Gene length/genome (%)	37.82%	34.91%	37.61%
Genome size (bp)	36,444,366	33,809,908	34,136,716

## Data Availability

The original contributions presented in this study are included in the article/Supplementary Materials. Further inquiries can be directed to the corresponding author.

## Supplementary Materials

The following supporting information can be downloaded at https://www.mdpi.com/article/10.3390/jof11050346/s1: Table S1: Statistics of AM whole-genome sequencing information characteristics.
